# Identification of a Novel LysR-Type Transcriptional Regulator in Staphylococcus aureus That Is Crucial for Secondary Tissue Colonization during Metastatic Bloodstream Infection

**DOI:** 10.1128/mBio.01646-20

**Published:** 2020-08-25

**Authors:** Michaela Groma, Sarah A. Horst, Sudip Das, Bruno Huettel, Maximilian Klepsch, Thomas Rudel, Eva Medina, Martin Fraunholz

**Affiliations:** a Chair of Microbiology, Biocenter, University of Würzburg, Würzburg, Germany; b Helmholtz-Zentrum für Infektionsforschung, Braunschweig, Germany; c Max Planck Genome Centre, Cologne, Germany; d Department of Fundamental Microbiology, University of Lausanne, Lausanne, Switzerland; e Helmholtz Institute for RNA-based Infection Research (HIRI), Würzburg, Germany; University of Nebraska Medical Center; Harvard Medical School

**Keywords:** *Staphylococcus aureus*, metabolic adaptation, secondary site infection, transcriptional regulation

## Abstract

Staphylococcus aureus is a common cause of bacteremia that can lead to severe complications once the bacteria exit the bloodstream and establish infection in secondary organs. Despite its clinical relevance, little is known about the bacterial factors facilitating the development of these metastatic infections. Here, we used an S. aureus transposon mutant library coupled to transposon insertion sequencing (Tn-Seq) to identify genes that are critical for efficient bacterial colonization of secondary organs in a murine model of metastatic bloodstream infection. Our transposon screen identified a LysR-type transcriptional regulator (LTTR), which was required for efficient colonization of secondary organs such as the kidneys in infected mice. The critical role of LTTR in secondary organ colonization was confirmed using an isogenic mutant deficient in the expression of LTTR. To identify the set of genes controlled by LTTR, we used an S. aureus strain carrying the LTTR gene in an inducible expression plasmid. Gene expression analysis upon induction of LTTR showed increased transcription of genes involved in branched-chain amino acid biosynthesis, a methionine sulfoxide reductase, and a copper transporter as well as decreased transcription of genes coding for urease and components of pyrimidine nucleotides. Furthermore, we show that transcription of LTTR is repressed by glucose, is induced under microaerobic conditions, and required trace amounts of copper ions. Our data thus pinpoints LTTR as an important element that enables a rapid adaptation of S. aureus to the changing host microenvironment.

## Abstract (precis)

Staphylococcus aureus is an important pathogen that can disseminate via the bloodstream and establish metastatic infections in distant organs. To achieve a better understanding of the bacterial factors facilitating the development of these metastatic infections, we used in this study a Staphylococcus aureus transposon mutant library in a murine model of intravenous infection, where bacteria first colonize the liver as the primary infection site and subsequently progress to secondary sites such as the kidney and bones. We identified a novel LysR-type transcriptional regulator (LTTR), which was specifically required by S. aureus for efficient colonization of secondary organs. We also determined the transcriptional activation as well as the regulon of LTTR, which suggests that this regulator is involved in the metabolic adaptation of S. aureus to the host microenvironment found in secondary infection sites.

## INTRODUCTION

Staphylococcus aureus is a common colonizer of the human skin and mucosal membranes without causing diseases. However, S. aureus can also cause a variety of severe infections such as sepsis, abscesses in deep tissues, and osteomyelitis after reaching sterile anatomic sites, for example, via the bloodstream (1). The emergence and spread of S. aureus strains resistant to multiple antibiotics (2) added to the remarkable capacity of S. aureus to evade elimination by the host immune defenses (3) and make this pathogen a formidable challenge for physicians. Despite extensive efforts made by the research community to uncover the pathogenic strategies of S. aureus, many aspects of the infection process remain unclear. In metastatic bloodstream infections, S. aureus disseminates from a primary site of infection such as skin abscesses, intravenous catheters, or surgical sites to secondary organs (4). Several virulence factors have been reported to mediate the interaction and/or extravasation of S. aureus through the endothelium, including the extracellular adherent protein Eap, wall teichoic acid, or fibronectin-binding proteins (5). After extravasation from the bloodstream to adjacent tissue, S. aureus must adapt to the physicochemical microenvironment and nutrient availability as well as to the local immune response in the new niches in order to survive, proliferate, and establish productive infection. The virulence mechanisms of S. aureus that facilitate these processes remain largely unknown. A deeper understanding of these pathogenic mechanisms can provide novel opportunities for therapeutic interventions in bacteremic patients.

We previously reported the remarkable plasticity of S. aureus to reprogram its expression of virulence factors during infection to adapt to the microenvironment and to the biological pressure imposed by the host (6, 7). In this study, we have used a S. aureus transposon insertion library coupled to deep sequencing of transposon insertion sites (Tn-Seq) to identify genes required by S. aureus to colonize and establish infection in secondary organs during *in vivo* infection. In general, Tn-Seq has become a very popular high-throughput technique that has been widely applied to identify genes and pathways that are important for infection for different pathogens (8–10). We and others have successfully applied this technique to identify novel factors involved in S. aureus pathogenicity (11, 12). Thus, in a previous study using a transposon mutant library generated with the staphylococcal strain 6850, we identified the *araC*-type transcriptional regulator repressor of surface proteins *rsp* as a pleiotropic virulence factors regulator during the initial stages of infection (11). Here, we screened this S. aureus transposon insertion library (11) in a well-established mouse model of metastatic bloodstream infection (13). Mice were infected with the mutant library, and the mutants recovered from the primary site of infection, the liver, as well as from the secondary infected organs, the kidneys in this case, were compared with those present in the original inoculum. The mutants underrepresented in the pool of colonizing bacteria in comparison with the original inoculum indicated the genetic determinants that are critical for optimal establishment of infection in secondary organs. A single gene was identified (RSAU_000852) that encoded a LysR-type transcriptional regulator (LTTR), whose mutants were specifically depleted in the bacterial pool recovered from the kidneys. An isogenic mutant in LTTR exhibited a significantly reduced capacity to survive in kidneys and tibiae compared to that of the wild type, thus corroborating the results of the Tn-Seq analysis. Functional characterization of LTTR indicated that this regulator may be required by S. aureus to rapidly adjust to the environmental conditions and nutrient availability in secondary organs.

## RESULTS

### Tn-Seq identifies a novel transcriptional regulator of S. aureus required for colonization of secondary organs.

To identify factors required for S. aureus colonization of secondary organs during bloodstream infection, mice were intravenously infected with 10^6^ CFU of a transposon mutant library of S. aureus strain 6850 (11), livers and kidneys were isolated from infected mice (*n* = 3) at 24 h after bacterial inoculation, and organ homogenates were plated onto blood agar. The numbers of bacteria at the time of sampling were 4.86 ± 0.69 log_10_ CFU in livers and 5.3 ± 0.7 log_10_ CFU in kidneys. Bacteria were collected from the plates and pooled, and enrichment or depletion of bacterial insertional mutants in kidneys and livers of infected mice was assessed by deep-sequencing of transposon insertion sites (Tn-Seq).

**FIG 1:**
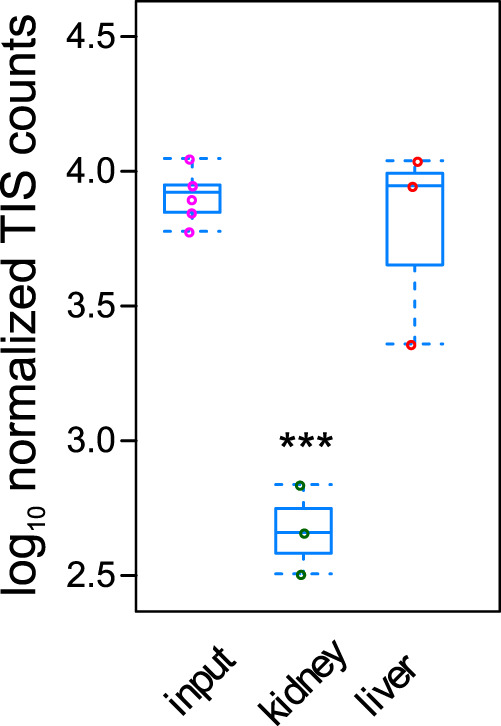
The frequency of S. aureus with mutation in RSAU_000852 is significantly lower in the bacterial pool recovered from infected kidneys than in the original inoculum. C57BL/6 mice (*n* = 3) were intravenously infected with a S. aureus 6850 transposon mutant library, and viable bacteria were recovered from liver and kidneys at 24 h of infection. Pools of the recovered bacteria and the respective inoculum (input) were analyzed by Tn-Seq, and counts of transposon insertion sites were compared by DESeq2. ***, *P* < 0.001.

Of the five genes (RSAU_000958 [*purM*], RSAU_000901, RSAU_000571 [*ltaA*], a hypothetical protein RSAU_000852, and RSAU_002542, a putative DNA-binding protein) whose mutants were significantly depleted in bacteria recovered from the kidneys in comparison to that in the inoculated mutant pool (Table 1), four were also found depleted in the liver: RSAU_000958, RSAU_000901, RSAU_000571, and RSAU_002542 (see Tables S4 to S6 in the supplemental material). Whereas *purM* and *ItaA* are genes with known functions and involved in pathways that are important for the physiology of S. aureus, such as purine biosynthesis and the cell wall, respectively, RSAU_000852 (NCTC8325 identifier [ID] SAOUHSC_00913), whose mutant was depleted only in the kidneys, was a novel gene with unknown function. Because this gene is homologous to a gene encoding a LysR-type transcriptional regulator, RSAU_000852 was termed LysR-type transcriptional regulator (LTTR). Mutants in LTTR were significantly depleted in bacteria recovered from kidneys (log_2_ fold change [log_2_FC], 3.95; *P* = 5.6 × 10^−7^), whereas read counts obtained from liver did not differ significantly from those of the inoculum (log_2_FC = 0.18, *P* = 0.84) (Fig. 1). Sequence alignments indicated that LTTR is highly conserved in S. aureus, where sequence variants share at least 96% identity on the amino acid level. In addition to S. aureus, homologues were also observed in the closely related species Staphylococcus schweitzeri and Staphylococcus argenteus, with slightly different gene variants being observed in *S. argenteus* (84% to 87% of amino acid sequence identity between *S. argenteus* and S. aureus proteins) (data not shown). Since the gene is highly conserved in S. aureus, we focused our efforts on the functional characterization of this novel regulator that seems to be critical for the fitness of S. aureus during colonization of secondary organs. For this purpose, we generated an isogenic knockout mutant within the S. aureus strain 6850 deficient in the expression of LTTR (Δ852). By genome sequencing, we excluded the introduction of secondary site mutations during the mutagenesis procedure, since the method involved selection of an integrative plasmid under non-permissive conditions (treatment at 42°C), which had been shown to result in mutations within the *sae* two-component system.

**TABLE 1:** List of genes identified based on the enrichment or depletion of the respective transposon mutant in murine kidneys with respect to that in the intravenously inoculated transposon mutant pool

Locus ID	log_2_FC^*a*^	Adj. *P* value^*b*^	Gene or annotation
RSAU_000958	−5.00	1.50E−04	*purM*
RSAU_000901	−4.11	1.24E−04	*ltaA*
RSAU_000571	−4.04	1.24E−04	Hypothetical protein
RSAU_000852	−3.95	5.60E−07	LTTR
RSAU_002542	−2.94	1.24E−04	DNA-binding protein, putative
RSAU_000494	3.72	2.75E−04	*rpoB*
RSAU_000940	3.74	2.40E−04	*atl*
RSAU_000222	3.96	1.24E−04	*lytM*
RSAU_000981	4.09	2.85E−04	*pdhD*
RSAU_001882	4.26	3.88E−04	*Gcp*
RSAU_000151	4.33	4.86E−04	Putative transporter, permease component transport system permease protein
RSAU_001970	4.40	2.62E−04	ATP-grasp domain protein
RSAU_002089	4.43	2.02E−04	*pbuG*
RSAU_000495	4.50	7.74E−04	*rpoC*
RSAU_001555	4.51	4.52E−04	*pyk*
RSAU_000943	4.53	6.32E−04	*nanE*
RSAU_002274	4.53	1.89E−04	*bar*
RSAU_000822	4.57	2.91E−04	Pyridine nucleotide-disulfide oxidoreductase family protein
RSAU_001560	4.81	1.24E−04	*dnaE*
RSAU_000844	4.84	1.24E−04	*addA*
RSAU_002334	5.00	7.83E−04	*pgcA*
RSAU_000006	5.04	1.76E−06	*gyrA*
RSAU_000735	5.04	8.10E−04	*hprK*
RSAU_000187	5.08	2.36E−04	*ptsG*
RSAU_000193	5.10	2.36E−04	*gutB*
RSAU_001351	5.14	1.24E−04	*ebpS*
RSAU_002484	5.15	5.55E−04	*manP*
RSAU_002525	5.16	3.05E−04	*hisZ*
RSAU_000259	5.17	9.20E−04	*nupC*
RSAU_000009	5.19	1.24E−04	*serS*
RSAU_000666	5.22	1.50E−04	Transporter anion-sodium symporter family protein
RSAU_002543	5.25	1.24E−04	*nixA*
RSAU_001219	5.28	2.50E−04	*guaC*
RSAU_000600	5.31	7.74E−04	*nupC2*
RSAU_002444	5.35	1.17E−04	Amino acid permease family protein
RSAU_000994	5.58	4.08E−07	*typA*
RSAU_000042	5.59	2.28E−04	Dienelactone hydrolase family protein
RSAU_002289	5.60	1.96E−04	*rpsP*
RSAU_001080	5.62	7.42E−04	*rluD*
RSAU_002278	5.70	4.86E−04	*cycA*
RSAU_000062	5.76	3.24E−05	67-kDa myosin-cross-reactive antigen
RSAU_001145	5.77	2.91E−04	*proS*
RSAU_001630	5.84	7.83E−04	Mannosyl-glycoprotein endo-beta-*N*-acetylglucosaminidase protein
RSAU_000638	5.94	1.24E−04	*vraG*
RSAU_000864	5.97	4.22E−04	*oppA*
RSAU_000268	5.98	6.89E−04	*yqiG*
RSAU_002020	6.21	2.02E−04	*htsB*
RSAU_000696	6.23	5.21E−04	*ptsH*
RSAU_000631	6.32	1.24E−04	*lipA*
RSAU_000427	6.50	2.40E−04	*metG*
RSAU_002217	6.51	9.28E−09	*rsp*
RSAU_002432	6.60	3.09E−04	Adenine nucleotide alpha hydrolases superfamily protein
RSAU_001217	6.78	9.15E−04	rpmG
RSAU_002292	7.11	3.22E−07	rimM
RSAU_000505	7.24	6.55E−05	capD

a Enrichment and depletion are indicated by positive and negative log_2_FC, respectively.

b Cutoff, adjusted (adj.) *P* values of ≤0.001.

### LTTR is important for S. aureus successful colonization of secondary infection sites.

**FIG 2:**
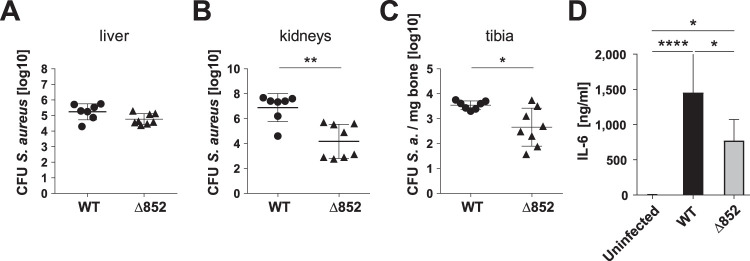
LTTR is required by S. aureus for successful infection of secondary organs. Mice were infected intravenously with WT (●) and Δ852 (▲), and CFU where determined in liver (A), kidneys (B) and tibiae (C) at 24 h of infection. Each symbol represents the data of an individual animal. Data are pooled from three independent experiments. (D) Serum levels of IL-6 in uninfected or in mice infected with either wild-type or Δ852 mutant strain at 24 h of infection. Each column represents the mean values ± standard deviations (SDs) from three independent experiments. *, *P* < 0.05; **, *P* < 0.01; ****, *P* < 0.001.

To validate the results of the transposon mutant screen, we compared the capacity of S. aureus Δ852 to establish infection in secondary organs after intravenous inoculation to that of the wild-type (WT) strain. Whereas CFU of WT and mutant strain recovered from livers did not differ significantly (Fig. 2A), the numbers of bacteria recovered from kidneys (Fig. 2B) and bones (Fig. 2C) were significantly lower in mice infected with the Δ852 strain than in those infected with the WT strain. The serum levels of the inflammatory cytokine interleukin 6 (IL-6) were significantly lower in mice infected with the Δ852 strain than in mice infected with the wild-type strain, further corroborating the attenuated phenotype of the Δ852 mutant (Fig. 2D). These results supported the requirement of LTTR for successful S. aureus infection of secondary organs during bloodstream infection.

### Characterization of the regulon of the novel LTTR.

**FIG 3:**
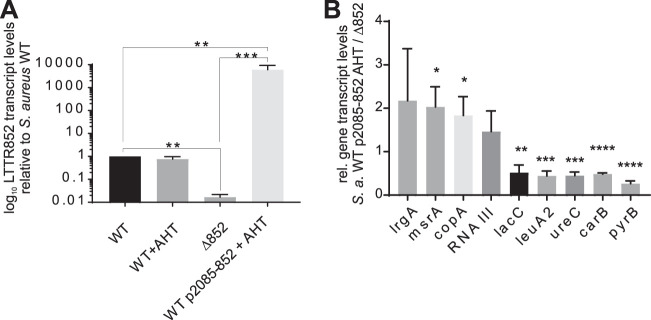
Validation of RNA-seq data by quantitative RT-PCR. (A) Expression level of LTTR (RSAU_000852) in S. aureus WT + AHT, Δ852 and WT p2085-852 + AHT relative to the expression in the WT strain after stimulation of LTTR expression with 200 ng/ml AHT for 1 h (*n* = 3). Notice the similar levels of LTTR expression in WT and WT + AHT and the almost undetectable level in Δ852. (B) Expression level of a set of genes in WT p2085-852 (+ AHT) relative to the expression in Δ852. Statistical analysis was performed using Student’s *t* test. *, *P* < 0.05; **, *P* < 0.01; ***, *P* < 0.001; ****, *P* < 0.0001.

To identify the regulon controlled by LTTR, S. aureus WT and S. aureus Δ852 were transduced with an anhydrous tetracycline (AHT)-inducible plasmid (14) carrying LTTR (WT_AHT and Δ852_AHT, respectively). The gene expression profile of S. aureus WT, Δ852, WT_AHT, and Δ852_AHT strains cultured in Trypticase soy broth (TSB) was analyzed using transcriptome sequencing (RNA-seq) after inducing LTTR expression by addition of 200 ng/ml of AHT for 1 h and the genes differentially expressed between the different strains were assessed by DESeq2 analysis. Several transcripts were found specifically up- and downregulated upon induction of LTTR (Table 2). Genes upregulated upon induction of LTTR expression included peptide methionine sulfoxide reductase *msrA2*, the components of a copper-translocation machinery *copA* and *copZ*, the genes of branched-chain amino acid biosynthesis *ilvD*, *ilvB*, and *leuA2*, and the holin-like murein hydrolase regulator *lrgA*. The genes with reduced expression after LTTR induction included members of the urease operon (*ureB*, *ureC*, *ureE*, and *ureF*), genes of pyrimidine biosynthesis pathway (*pyrB*, *pyrC*, and *carB*), the gene coding for tagatose-6-phosphate kinase (*lacC*) and phosphotransferase system (PTS) lactose-specific transporter subunit IIBC (*lacE*). The upregulation of LTTR in Δ852_AHT after treatment with AHT was validated by reverse transcription-PCR (RT-PCR) (Fig. 3A). Furthermore, the expression of a set of genes found in the RNA-seq data either upregulated (*msrA*, *copA*, *lrgA*, and RNAIII) or downregulated (*leuA2*, *ureC*, *leuA1*, *carB*, *lacC*, and *pyrB*) after LTTR induction in WT-AHT versus that in the Δ852 strain were also confirmed by RT-PCR (Fig. 3B).

**TABLE 2:**
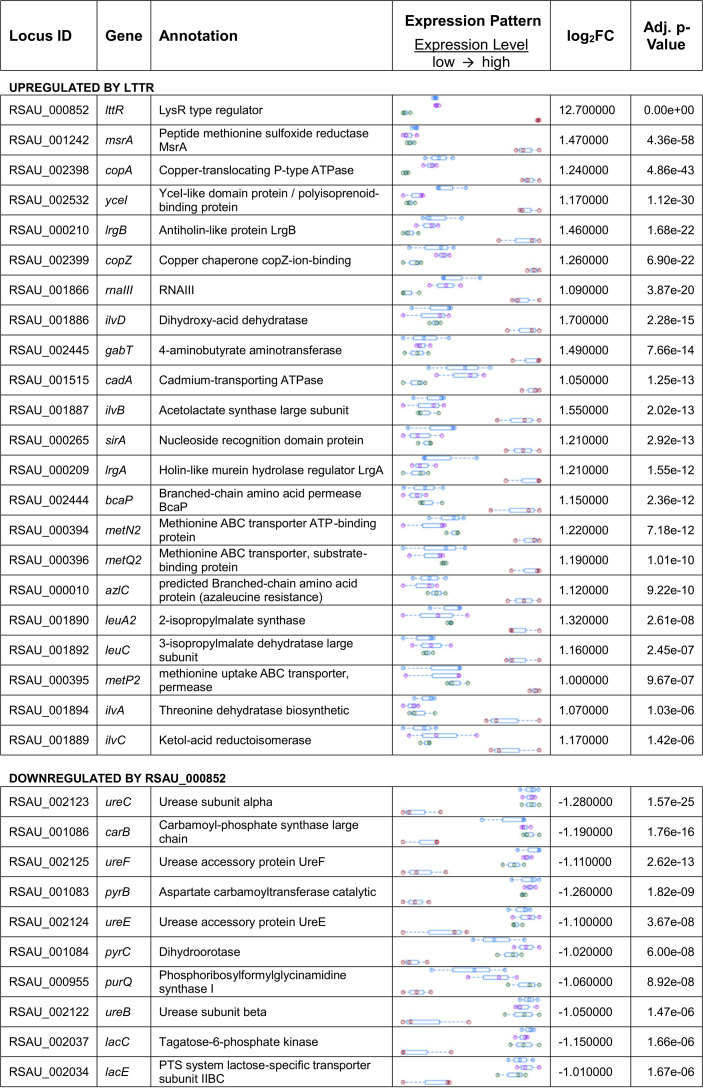
Regulated genes upon induced expression of LTTR^*a*^

a The expression pattern is color coded: blue, S. aureus WT; magenta, WT treated with 200 ng/ml AHT; green, S. aureus Δ852; red, S. aureus pI852, LTTR expression induced with 200 ng/ml AHT.

Using the genes’ ortholog identifiers for strain NCTC8325, we found that the potential LTTR target genes were significantly enriched in gene ontology (GO) terms for the biological processes “branched-chain amino acid biosynthetic process” (GO:0009082), “urea metabolic process (GO:0019627), and “*de novo* uridin monophosphate (UMP) biosynthetic process” (GO:0044205) (see Table S3).

### Transcription of LTTR is enhanced under infection-mimicking conditions.

To investigate under which conditions LTTR is expressed by S. aureus, a promoter fusion with a codon-adapted green fluorescent protein (GFP) (15) was generated yielding the plasmid p2085-Pr852-GFP that was introduced into S. aureus WT. Because oxygen levels at sites of tissue inflammation may be relatively low as a result of impaired perfusion (16), we recorded bacterial growth and GFP fluorescence levels under aerobic and microaerobic conditions. No differences were observed in bacterial growth under aerobic or microaerobic conditions (see Fig. S1A). However, promoter activity was stronger in bacteria grown under microaerobic conditions than in those grown under aerobic conditions (Fig. S1B and C). We therefore determined the levels of transcription of LTTR in S. aureus WT grown in TSB under aerobic and microaerobic conditions and found the transcription levels of the gene to be significantly higher under microaerobic conditions than when grown in the presence of oxygen (Fig. S1D).

10.1128/mBio.01646-20.1 FIG S1 Microaerophily has a positive effect on the promotor expression of LTTR. (A) Kinetics of S. aureus WT growth determined by OD_600_ under microaerobic (lighter color) or under aerobic (darker color) conditions (*n* = 3). (B) Mean fluorescence of the GFP linked to the LTTR promoter region in S. aureus WT under microaerobic (lighter color) or under aerobic (darker color) conditions. (C) Ratio of GFP to OD (GFP/OD) is higher under microaerobic conditions. (D) Relative transcription level of LTTR in S. aureus WT grown in TSB for 6 h under aerobic or microaerobic conditions. (E) GFP/OD ratios of wild-type S. aureus 6850 in CDM under microaerobic (light grey) or aerobic conditions (dark grey). Statistical analysis was performed using Student’s *t* test. **, *P* < 0.01. Download FIG S1, PDF file, 0.1 MB. Copyright © 2020 Groma et al. 2020 Groma et al. This content is distributed under the terms of the Creative Commons Attribution 4.0 International license.

**FIG 4:**
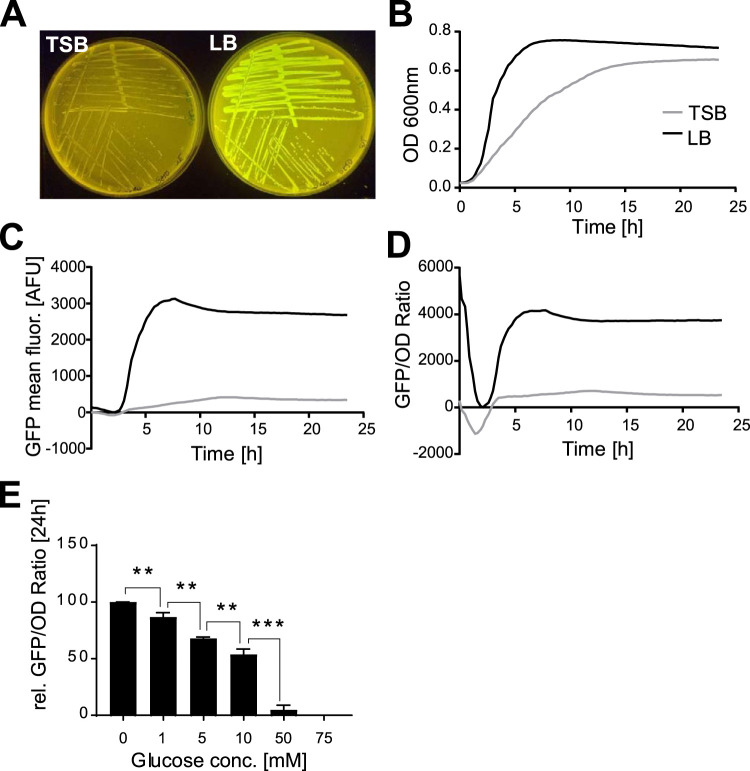
Transcription of LTTR is repressed by glucose. (A) GFP fluorescence of S. aureus 6850 harboring the LTTR promoter reporter plasmid Pr852-GFP is less intense in TSB agar containing 2.5 g/liter glucose (left) than in glucose-free LB agar (right). (B to D) Kinetics of S. aureus *6850* Pr852-GFP growth determined over a 24 h period under microaerobic conditions in either glucose-containing TSB or glucose-free LB medium. The changes in OD_600_ are shown in panel B, the mean GFP fluorescence in panel C and the GFP/OD ratios in panel D. (E) GFP/OD ratios after 24 h of culture of the promoter reporter strain grown under microaerobic conditions in CDM supplemented with different glucose concentrations showing a concentration-dependent repression of the LTTR promoter by glucose. Each kinetics experiment was performed in triplicates (*n* = 3). Statistical analysis was performed using Student’s *t* test. **, *P* < 0.01; ***, *P* < 0.001.

Interestingly, during the process of generating the GFP reporter strain, we observed that S. aureus Pr852-GFP plated on LB agar without glucose exhibited more intense GFP fluorescence than when it was grown in TSB agar containing 2.5 g/liter glucose (Fig. 4A). Similarly, we observed difference during growth in broth as shown by the slightly faster bacterial growth in glucose-free LB medium than in TSB containing 2.5 g/liter glucose. The mid-log phase was reached after 3 h in LB and after 7 h of culture in TSB (Fig. 4B). Again, promoter activity of Pr852 was drastically increased in LB in comparison to that in TSB (Fig. 4C and D). To investigate the repression by glucose, we grew WT and mutant bacteria in chemically defined medium (CDM) containing various concentrations of glucose ranging from 0 to 75 mM. We recorded bacterial growth as well as Pr852 promoter activity over a period of 24 h (Fig. 4E). Addition of 10 mM glucose resulted in ∼50% reduction of promoter activity, which was almost completely abolished at 50 mM and 75 mM glucose. Our results hereby indicated that the promoter of LTTR was repressed by glucose.

**FIG 5:**
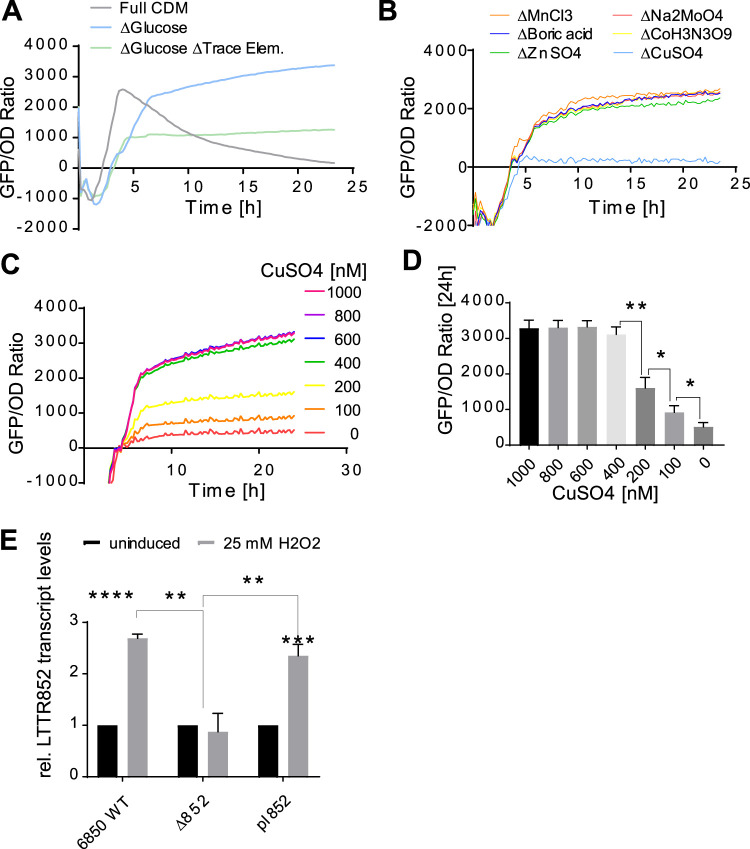
LTTR promoter activity is observed under infection-mimicking conditions and requires copper ions. (A) GFP/OD ratios of wild-type S. aureus 6850 growing under microaerobic conditions in full CDM (light gray), CDM lacking glucose (blue), or lacking both glucose and trace elements (green). (B) Microaerobic growth in glucose-free CDM lacking single constituents of the trace element mixture identifies a copper requirement for promoter activity. (C) Copper-dependent dose response of the LTTR promoter is observed during microaerobic growth in glucose-free CDM and increasing amounts of copper sulfate ranging from 0 to 1,000 nM. (D) 400 nM copper sulfate restores full promoter activity, whereas lower concentrations show a significant dose-dependent decrease in promoter activities. (E) The LTTR promoter is induced by 25 mM hydrogen peroxide. Relative transcription level of the different strains under uninduced (black bars) conditions or after treatment with 25 mM H_2_O_2_ for 1 h (gray bars). Each experiment was performed in triplicates (*n* = 3). Statistical analysis was performed using Student’s *t* test. *, *P* < 0.05; **, *P* < 0.01; ***, *P* < 0.001; ****, *P* < 0.0001.

To investigate additional nutritional requirements for LTTR transcription, we used CDM and determined LTTR promoter activity by the levels of GFP fluorescence. We first corroborated that promoter activity was enhanced under microaerobic conditions in CDM (Fig. S1E) and increased in the absence of glucose (Fig. 5A). We next determined LTTR promoter activity in CDM without glucose and omitting other components of the defined medium. Upon omission of the trace metal mixture, we identified reduced LTTR activity (Fig. 5A). We thus omitted single constituents of the trace mixture, including boric acid, manganese(II) chloride, zinc sulfate, sodium molybdate, cobalt nitrate, or copper sulfate (Fig. 5B; see Fig. S2A to F). LTTR promoter activity was abolished only in CDM without copper sulfate but showed a dose-dependent increase upon addition of different concentrations of copper sulfate and was fully reconstituted by addition of 400 nM copper sulfate (Fig. 5C and D). Similarly, the relative transcription level of LTTR in strains grown in CDM without glucose (Δglucose) compared to that in CDM additionally lacking copper sulfate (CDM Δglucose Δcopper) demonstrated that the transcription level of LTTR is significantly higher in S. aureus WT (threshold cycle difference [ΔΔ*C_T_*] = 2.375 ± 0.1737, *P* value = 0.0014) as well as in Δ852 bacteria carrying the complementation plasmid pI852 (ΔΔ*C_T_* = 2.527 ± 0.1795, *P* value = 0.0010) when copper sulfate was present in the medium (Fig. S2G).

10.1128/mBio.01646-20.2 FIG S2 LTTR 852 Promotor activity under microaerobic conditions in CDM without glucose and trace elements illustrates the requirement of copper. GFP/OD ratio of S. aureus p852 GFP in glucose-free CDM lacking either boric acid (A), manganese(II) chloride (B), zinc sulfate (C), sodium molybdate (D), cobalt(II) nitrate (E), or copper sulfate (F). (*G*) Significantly higher expression of LTTR in the wild type and complement is observed when strains are grown in CDM Δglucose in the presence of copper sulfate. Depicted are relative transcription levels of LTTR in wild type (WT) and complement (pl852) strains with or without copper sulfate. Mutant expression levels were set to 1. Statistical analysis was performed using Student’s *t* test. **, *P* < 0.01. Download FIG S2, PDF file, 0.1 MB. Copyright © 2020 Groma et al. 2020 Groma et al. This content is distributed under the terms of the Creative Commons Attribution 4.0 International license.

Our RNA-seq data after induced expression of LTTR identified the oxidative stress response gene *msrA2* as a target gene. We therefore hypothesized that LTTR may be important for S. aureus upon encounter of oxidative stress in the host. To test this hypothesis, we compared the levels of LTTR gene RSAU_000852 transcription between S. aureus WT, the Δ852 mutant, and the complemented mutant grown under normal growth conditions and under oxidative stress conditions generated after a pulse with 25 mM hydrogen peroxide for 1 h. Transcriptional expression of the well-known oxidative stress response genes *dps* and *sodA* was used to corroborate that peroxide exposure induced an oxidative stress response in S. aureus (see Fig. S3). Transcription of RSAU_000852 was induced in the WT (2.69 [± 0.05]-fold induction; *P* value < 0.0001) and the complement strain (2.35 [± 0.13]-fold induction; *P* value = 0.0004) under H_2_O_2_ stress, which was absent in the Δ852 mutant (Fig. 5E).

10.1128/mBio.01646-20.3 FIG S3 LTTR transcription is induced by peroxide stress: relative transcription levels of *dps* and *sodA* show induction of oxidative stress. Relative transcription level of the S. aureus 6850 WT, the LTTR mutant (Δ852), and the complementation strain (pI852) under normal growth conditions (black bars) or after treatment with 25 mM H_2_O_2_ for 1 h (grey bar). General stress protein *dps* (A) and superoxide dismutase *sodA* (B) are induced demonstrating a transcriptional response of S. aureus to reactive oxygen species. *, *P* < 0.05; **, *P* < 0.01; ***, *P* < 0.001; ****, *P* < 0.0001. Download FIG S3, PDF file, 0.1 MB. Copyright © 2020 Groma et al. 2020 Groma et al. This content is distributed under the terms of the Creative Commons Attribution 4.0 International license.

## DISCUSSION

After invasion of the host, pathogens need to rapidly adjust to the different tissue microenvironments encountered during the infection process in order to successfully establish infections. This involves sensing local conditions, including nutrient availability and resident host immune defenses, and adjusting their gene expression and virulence functions accordingly. Thus, within the infection niches in the host, pathogens need to overcome barriers that sharply limit their population size, the so-called bottlenecks. For example, after intravenous inoculation, S. aureus quickly accumulates within the liver, which constitutes the first infection niche of the host. Within the liver, a high proportion of the inoculated bacteria are sequestered by Kupffer cells, which are liver macrophages and represent the first infection bottleneck. Although a proportion of the bacteria sequestered within Kupffer cells are efficiently killed, a subpopulation of internalized S. aureus bacteria has been shown to survive and even multiply in the phagosomes and eventually kill the host cells (17). There, neutrophils take up the bacteria and may reenter circulation, thereby disseminating the pathogen from the liver to secondary infection sites (18). Thus, from the first niche in the liver, S. aureus disseminates to secondary infection niches such as the bones and kidneys, where distal abscesses are formed (6). It is thought that S. aureus requires different subsets of virulence factors to establish infection in the various niches it may encounter within the host.

### Tn-Seq identified a niche-specific transcription factor.

We hence used a transposon mutant library in S. aureus 6850 (19) to discover virulence factors critical for S. aureus to establish infection in secondary infection niches. Mice were intravenously inoculated with the transposon library and Tn-seq was applied to bacteria recovered from the primary infection site at the liver as well as from secondary infection sites at the kidneys to catalog the insertion mutants compared to the infection inoculum. Several genes were identified whose mutants were specifically depleted or enriched in either liver or kidney or in both organs of mice after experimental intravenous infection (Table 1; see also Tables S5 and S6 in the supplemental material).

Interestingly, the lists obtained by Tn insertion site sequencing do not contain classical (pore-forming) toxins such as staphylococcal alpha toxin or leukocidins, illustrating that the sequencing strategy would benefit from mutant libraries with higher complexities (12); however, the strategy is mostly limited by the strong selective effects. For instance, we initially tried to extend the Tn-Seq strategy to tibiae; however, the recovered number of bacteria was too low for analysis, thereby illustrating the strength of the bottleneck effects (data not shown).

We found only the RSAU_000852 mutant to be specifically depleted in secondary infection sites in kidneys in comparison with the original inoculated pool of mutants as well as with those mutants recovered from primary infection sites at the liver. These results indicate that expression of RSAU_000852 may be of critical importance for S. aureus to establish infection specifically in secondary niches within the host. This was corroborated by intravenous infection of mice with the seamless Δ852 deletion mutant deficient in the expression of RSAU_000852. Whereas the Δ852 mutant exhibited a significantly lower capacity to establish infection in kidneys and tibiae than WT bacteria, the number of Δ852 mutant bacteria recovered from liver was comparable to that of the WT strain. Furthermore, mice infected with the Δ852 mutant also exhibited significantly lower serum levels of the inflammatory cytokine IL-6 than those infected with the WT strain, which correlates with the attenuated phenotype of the mutant strain.

### Characterization of the novel LTTR.

Homology search identified a LysR-type transcriptional regulator (LTTR) as the product of RSAU_000852. LTTRs are known as the largest family of prokaryotic DNA-binding proteins, with 800 members identified on the basis of their amino acid sequence (20). They can act as either repressors or activators of single or operonic genes (21, 22). LTTRs regulate numerous genes whose products are involved in important bacterial functions such as metabolism, motility, cell division, quorum sensing, virulence, and oxidative stress response to name a few (23–31). Four LTTRs have been identified so far in S. aureus, including CidR, HutR, GltC, and CcpE (32–34). In this study, we have identified a new LTTR in S. aureus 6850 encoded by RSAU_000852, with unknown function and whose expression was rarely reported. For example, some studies have reported that RSAU_000852 was 2.94-fold downregulated in the presence of 0.01 μM linoleic acid (35) and 3.75-fold induced in strain COL exposed to 1 mM diamide (36). In other studies, however, where networks of gene regulation were investigated in S. aureus strain HG001 under 106 different conditions, the NCTC82325 LTTR_852 homolog, SAOUHSC_0913, was never found to be expressed under any of the conditions tested (37).

By using AHT-inducible expression of LTTR in S. aureus 6850 and subsequent RNA-seq, we established potential targets of the transcriptional regulator (Table 2). Genes found upregulated upon induction of LTTR expression included those encoding peptide methionine sulfoxide reductase *msr*A2 and components of a copper-translocation machinery, *copA* and the copper chaperone *cop*Z (38), which are involved in copper efflux, as well as genes encoding factors involved in branched-chain amino acid biosynthesis such as *ilvD* (dihydroxy-acid dehydratase), *ilvB*, *leuA2*, and the holin-like murein hydrolase regulator *lrgA*. Oxidation of methionine by the activity of methionine sulfoxide reductase can destroy protein structure or affect protein function (39). There are four methionine sulfoxide reductases harbored by S. aureus, of which three possess MsrA functionality and a single gene encoding MsrB (40). Interestingly, the operon encoding *msrA1* and *msrB* was found to be upregulated in S. aureus recovered from experimental animals during acute and chronic infection in comparison to S. aureus grown in medium *in vitro* (6). Our data suggest, however, that LTTR is specifically upregulating *msrA2*. Among the well-studied LTTRs is OxyR, which is responsible for the oxidative stress response against H_2_O_2_ in Escherichia coli and Salmonella enterica serovar Typhimurium (41). Peroxide is, for instance, produced as an antibacterial agent by many cells of the innate immune system. Hence, LTTR may be important for S. aureus upon encountering oxidative stress in the host.

Isopropylmalate synthase 2 encoded by *leuA2* is involved in the first step of the pathway of l-leucine biosynthesis. *leuA2* as well as other genes of the branched-chain amino acid (BCAA) biosynthesis machinery (*leuC*, *leuD*, *leuB*, and *ilvE*) and *bcaP*, a BCAA transporter (42), have been identified as target genes of LTTR by RNA-seq. Another virulence-associated BCAA transporter, *brnQ2* (RSAU_000252), was reported to be upregulated in S. aureus during chronic infections, suggesting CodY derepression and emphasizing the importance of branched-chain amino acid metabolism for S. aureus survival within the host (6). *brnQ1*, *brnQ2*, or *brnQ3* and other CodY targets, however, were not significantly regulated in our data set, suggesting that a CodY-independent induction of BCAA biosynthesis may exist in S. aureus. It has to be noted that transcription of LTTR was not derepressed in a *codY* mutant (43), although the promoter region of LTTR (within the intergenic region of the upstream gene *clpB* and RSAU_000852) was highly enriched in CodY chromatin precipitation analyses (43).

Genes with reduced expression after LTTR induction included members of the urease operon *ureB*, *ureC*, *ureE*, and *ureF* as well as genes of pyrimidine biosynthesis, *pyrB*, *pyrC*, and *carB*, suggesting that the *de novo* UMP biosynthesis pathway is repressed by LTTR. Urease is part of the of S. aureus response to acid shock and recently was shown to be required for bacterial persistence in murine kidney infection (44). *lacC* (tagatose-6-phosphate kinase) and the lactose-specific compound *lacE* (PTS lactose-specific transporter subunit IIBC) (45) were also found repressed by the LTTR. LacC is involved in synthesis of d-glyceraldehyde 3-phosphate and glycerone phosphate from d-tagatose 6-phosphate, and LacE is part of the phosphoenolpyruvate-dependent sugar phosphotransferase system (PTS; LacEF). Increased PTS expression has been hypothesized to facilitate the pathogenesis of S. aureus in mastitis (46) and may also facilitate S. aureus adaptation to secondary organs during bloodstream infection as shown in this study.

Overall, expression of potentially LTTR-regulated genes under induction conditions was not very high, although significant. We hypothesize that the lack of the specific coinducer that LysR-type transcriptional regulators usually require for their activity is responsible for this effect. Since LTTRs require coinducers for DNA binding or multimerization, we assume that the concentration of the hitherto unknown coinducer of LTTR was limiting under the conditions tested. LTTRs comprise an N-terminal helix-turn-helix (HTH) motif and a C-terminal LysR-substrate-binding region (20). Different coinducers with structural diversity (e.g., amino acid derivates, aromatics, ions, and various aliphatics) have been shown to be involved in the stimulation of various LTTRs (20). For example, the well-characterized LTTR ToxR of Burkholderia glumae activates local quorum sensing via toxoflavin (27). In rare cases, the coinducer operates as an activator and repressor such as α-methylene-γ-butyrolactone during pilus and capsule synthesis in Neisseria meningitidis (47). Certainly, not all cofactors are known; for example, for YofA involved in the cell division in Bacillus subtilis (30) and a Yersinia pestis corepressor (48), the cofactors remain unidentified. Further experiments will be required to identify the coinducer(s) of LTTR. The decreased fitness of a RSAU_000852 mutant in mouse kidneys and tibiae leads us to hypothesize that niche-specific molecules may account for full activity of the LTTR protein.

Regarding the microenvironments in which LTTR is induced, our data show that LTTR promoter activity specifically requires microaerobic and glucose-free conditions as well as low concentrations of copper ions. Kidneys are involved in glucose homeostasis through processes of gluconeogenesis, glucose filtration, reabsorption, and consumption (49). Modeling glucose metabolism in kidneys identified anaerobic glucose metabolism in the inner medulla, owing to the limited blood flow and low tissue oxygen tension (50). Thus, microenvironments exist in the kidneys that are compatible with promoter activity of RSAU_000852.

Furthermore, we identified copper as a limiting factor for the activation of LTTR transcription. Copper is a cofactor essential for many enzymes involved in a multitude of cellular functions of both host and pathogen, such as cellular respiration, iron transport, and free radical scavenging (51–53). Copper also serves as antibacterial agent and is pumped into phagosomes of macrophages in order to kill bacteria. S. aureus, in turn, possesses copper efflux pumps, which are involved in survival under high-copper stress. Recently, two novel genes were identified, *copB* and *copL* (54). *copB* functions in copper export and displayed genetic synergy with *copA*. The function of *copL* is independent of *copA* and *copB*, it is a membrane bound and surface-exposed lipoprotein that binds up to four Cu ions. Our data suggest that LTTR_852 not only is transcriptionally activated by low concentrations of copper ions but also is involved in *copAZ* expression and thereby can modulate the copper response of S. aureus.

LTTR promoter activity can be seen in multiple genetic backgrounds of S. aureus (see Fig. S4 in the supplemental material) Interestingly, copper depletion had no effect on the LTTR promoter activity in USA 300 JE2. USA 300 strains carry a copper resistance locus (*copXL*), uniquely associated with the staphylococcal cassette chromosome *mec* elements (SCC*mec*s) not found in other S. aureus lineages (55) (Fig. S4). *copL* encodes a membrane-bound and surface-exposed lipoprotein that binds up to four Cu^+^ ions (54) and, therefore, may supply copper to the bacterium after been transferred to a copper-depleted growth medium.

10.1128/mBio.01646-20.4 FIG S4 LTTR promoter activity in different S. aureus strains in CDM under various conditions. GFP/OD Ratios of S. aureus 6850 (A), USA 300 JE2 (B), Cowan I (C), and RN4220 (D) in CDM under microaerobic conditions (black), CDM without glucose under microaerobic (green), and aerobic conditions (red), as well as in medium without glucose and copper sulfate (blue). All experiments were performed in triplicates (*n* = 3). Download FIG S4, PDF file, 0.2 MB. Copyright © 2020 Groma et al. 2020 Groma et al. This content is distributed under the terms of the Creative Commons Attribution 4.0 International license.

RNA-seq after induced expression of LTTR suggested the oxidative stress response gene *msrA* as a target gene. We therefore hypothesized that LTTR may be important for S. aureus upon encounter of oxidative stress in the host (see above). Interestingly, we show induction of the LTTR gene RSAU_000852 under H_2_O_2_ stress, which is absent in the Δ852 mutant yet reinstated by supplying the locus in *trans*.

Taken together, our data show that the LTTR encoded by RSAU_000852 is a transcriptional regulator required in specific host niches for the efficient establishment of infection. Within this niche, glucose and oxygen are limited, copper ions must be present, and reactive oxygen concentrations may be elevated, possibly due to recruited immune cells.

A limitation of the study is that we conducted Tn-Seq in a S. aureus pool of mutants recovered from liver and kidneys of intravenously infected animals. We identified the regulator LTTR as important for colonization of secondary organs (kidneys and bones) but not for establishment of infection in the primary site of infection at the liver. Since we harvested the infected tissues at a single time point very early during infection (24 h), we cannot exclude a potential role of LTTR for bacterial survival in the liver at later times of infection.

In summary, we identified in this study a LysR-type transcriptional regulator in S. aureus that is required for bacterial colonization of secondary infection sites. We unveiled a complex dependency of transcriptional activation of the regulator gene and, by inducible expression, identified a set of potentially regulated genes, which includes genes required to cope with oxidative stress and for growth under low-oxygen and glucose-free conditions, such as those found in host tissues. Hence, we hypothesize that the LTTR may play an important role in metabolic adaptation of S. aureus to local infection sites in the host. Thus, therapeutic targeting of the novel regulatory factor identified in this study in patients with S. aureus bacteremia may result in reduced bacterial fitness and offer options for improving disease outcome.

## MATERIALS AND METHODS

### Mice and infection model.

Pathogen-free 10-week-old female C57BL/6 mice (20.5 ± 0.8 g body weight) were purchased from Harlan-Winkelmann (Envigo, The Netherlands). Mice were infected intravenously with 10^6^ CFU of S. aureus strain 6850 in 100 ml of phosphate-buffered saline (PBS) via a lateral tail vein. For determination of bacterial numbers in organs, mice were killed by CO_2_ asphyxiation at a specified time after bacterial inoculation, and organs were removed and homogenized in PBS. Serial 10-fold dilutions of organ homogenates were plated on blood agar plates. Bacterial colonies were counted after incubation at 37°C for 24 h and calculated as CFU per organ or per mg of bone. Animal experiments were performed in strict accordance with the German regulations of the Society for Laboratory Animal Science (GV-SOLAS) and the European Health Law of the Federation of Laboratory Animal Science Associations (FELASA), and animals were excluded from further analysis if killing was necessary according to the humane endpoints established by the ethical board. All experiments were approved by the ethical board Niedersächsisches Landesamt für Verbraucherschutz und Lebensmittelsicherheit, Oldenburg, Germany (LAVES; permit number 33.9-42502-04-13/1195).

### ELISA.

The concentration of IL-6 in serum of infected mice was determined by enzyme-linked immunosorbent assay (ELISA) according to the manufacturer’s recommendations (BD Biosciences).

### Bacterial culture.

S. aureus strains (see Table S1 in the supplemental material) were grown in Trypticase soy broth (TSB), lysogeny broth (LB), or chemically defined medium (CDM) using appropriate antibiotics. The Tn-Seq inoculum was prepared by growing the strains in brain heart infusion (BHI) to an optical density (OD) of 0.5 before they were washed with PBS, aliquoted, and stored at −80°C.

10.1128/mBio.01646-20.5 TABLE S1 Bacterial strains and plasmids used in this study. Download Table S1, PDF file, 0.2 MB. Copyright © 2020 Groma et al. 2020 Groma et al. This content is distributed under the terms of the Creative Commons Attribution 4.0 International license.

CDM contained final concentrations of 1× basic medium (12.5 mM Na_2_HPO_4_·2H_2_O, 10 mM KH_2_PO_4_, 1.65 mM MgSO_4_, 9.25 mM NH_4_Cl, 8.5 mM NaCl), 75 mM glucose, 0.142 mM sodium citrate tribasic dihydrate, and 1 mM each amino acid except for tyrosine (0.1 mM). The trace element mix A5 was purchased from Sigma-Aldrich (number 92949) and was supplemented with FeCl_3_ in NaOH and 0.1 μM NiCl_2_. Vitamin mix contained final concentrations of 0.29 μM 4-aminobenzoic acid, 0.29 μM thiamine hydrochloride, 0.21 μM Ca-d-pantothenic acid, 0.036 μM cyanocobalamin, 0.04 μM d-biotin, 0.81 μM nicotinamide, 0.62 μM pyridoxine hydrochloride, and 0.2 μM riboflavin.

For induction of LTTR expression by anhydrous tetracycline, overnight cultures were diluted in 10 ml fresh TSB containing 200 ng/ml anhydrous tetracycline and were incubated at 37°C at 750 rpm for 1 h. Bacteria were harvested and snap-frozen in liquid nitrogen.

### Promoter activity assays.

Promoter activities during bacterial growth were assessed by monitoring GFP fluorescence (excitation, 488 ± 9 nm; emission, 518 ± 20 nm) as well as optical density (at 600 nm) using a Tecan Infinite M200 multiplate reader. For this, bacteria were grown in either TSB, LB, CDM, or CDM without glucose overnight at 37°C at 180 rpm in the presence of 20 μg/ml chloramphenicol. If medium exchange was necessary, overnight cultures where washed once with PBS and were resuspended in the corresponding medium. Bacterial suspensions with an OD of 0.1 were added to a 48-well microtiter plate (400 μl/well) and further cultured 6 h or 24 h under aerobic and microaerobic conditions. A microaerobic atmosphere was established by sealing the microtiter plates with adhesive foil and thereby limiting oxygen availability. Microaerobic conditions were confirmed by the relative induction of the gene encoding formate acetyltransferase (*pflB*), which is expressed only under low oxygen conditions (56). Absorbance and GFP fluorescence were recorded every 10 min using a Tecan Infinite M200 multiplate reader and were evaluated using Microsoft Excel.

### Staphylococcus aureus Tn-Seq.

Pooled mariner transposon mutant libraries were generated as previously described (11, 57). Bacterial DNA was isolated from the infection inoculum and from the bacterial colonies derived from infected tissues as previously described (11). The libraries were sequenced on the Illumina Hi-Seq 2500 platform with the transposon-specific oligonucleotide primer Himar1-Seq (11). Illumina adapter sequences were removed via cutadapt version 1.2.1 (58). The reads also were filtered for size (>16 bp) and to contain the transposon inverted terminal repeat (ITR) and were mapped to the Staphylococcus aureus 6850 genome (RefSeq accession NC_022222.1) by Bowtie2 v2.1.0 (59). Identification of depleted and enriched mutants was performed via DESeq2 version 1.6.2 (60).

### Cloning procedures.

Chromosomal DNA of S. aureus was prepared using the QIAprep Spin miniprep kit from Qiagen with the following modification: after resuspension of the bacteria in buffer P1, we added 50 μl lysostaphin and incubated at 37°C for 30 min (750 rpm).

For deletion of RSAU_000852 in S. aureus 6850, regions approximately 1 kb upstream and downstream were amplified by PCR using primers attB1-852-up-F and 852-up-R-SacII as well as 852-down-F-SacII and attB2-852-down-R, respectively (Table S2). The PCR product was cloned into pKOR1, and a markerless targeted gene deletion was then generated as previously described (61). To exclude relevant secondary site mutations, we sequenced the genome of the mutant (https://www.eurofinsgenomics.eu/). Sequence reads were mapped to the genome sequence of the wild-type S. aureus 6850 genome (RefSeq accession NC_022222.1).

10.1128/mBio.01646-20.6 TABLE S2 Oligonucleotides used in this study. Download Table S2, PDF file, 0.10 MB. Copyright © 2020 Groma et al. 2020 Groma et al. This content is distributed under the terms of the Creative Commons Attribution 4.0 International license.

10.1128/mBio.01646-20.7 TABLE S3 Enrichment in GO terms as determined by https://string-db.org/. Download Table S3, PDF file, 0.1 MB. Copyright © 2020 Groma et al. 2020 Groma et al. This content is distributed under the terms of the Creative Commons Attribution 4.0 International license.

10.1128/mBio.01646-20.8 TABLE S4 Gene mutants identified enriched or depleted in murine liver after intravenous infection (cutoff, adj. *P* values of ≤0.0005). Download Table S4, PDF file, 0.1 MB. Copyright © 2020 Groma et al. 2020 Groma et al. This content is distributed under the terms of the Creative Commons Attribution 4.0 International license.

10.1128/mBio.01646-20.9 TABLE S5 S. aureus transposon mutants found either enriched or depleted in kidneys and/or livers of mice after intravenous infection by using TN-seq. Counts of sequenced transposon insertion sites were compared with the counts obtained from the infection inoculum. By comparing the inoculum to the recovered samples, genes which are lost in a specific tissue are represented by negative log_2_FC and vice versa. Download Table S5, PDF file, 0.6 MB. Copyright © 2020 Groma et al. 2020 Groma et al. This content is distributed under the terms of the Creative Commons Attribution 4.0 International license.

10.1128/mBio.01646-20.10 TABLE S6 S. aureus transposon mutants identified by Tn-seq with sequence reads enriched (positive log_2_ fold changes [log_2_FC]) or depleted (negative log_2_FC) in either kidneys or livers of mice 24 h after intravenous infection compared to the infection inoculum (adjusted *P* value cutoff of 0.05). Shown are the open reading frame (ORF) ID of strain 6850 as well as its homolog within strain NCTC8325, gene assignment, annotation, log2FC, and adjusted *P* value of reads obtained from bacteria recovered from infected kidneys and livers. Genes that were identified as infection relevant in a previous study (67) are indicated. Download Table S6, XLSX file, 0.1 MB. Copyright © 2020 Groma et al. 2020 Groma et al. This content is distributed under the terms of the Creative Commons Attribution 4.0 International license.

The RSAU_000852 ORF was amplified from S. aureus genomic DNA using oligonucleotides 852-NotI-F and 852-BamHI-R (Table S2). Plasmid p2085-852, allowing for anhydrous tetracycline-inducible expression of RSAU_000852, was generated by restricting the PCR product as well as the vector, p2085 (62), with BamHI and NotI and subsequent ligation of vector and insert. The resulting plasmid was transformed into E. coli DH5α and plated on LB agar supplemented with ampicillin. The plasmid isolated from E. coli was sequenced, propagated within the cloning strain S. aureus RN4220, and transformed into electrocompetent S. aureus 6850.

For generation of the LTTR promoter (pr852) activity construct, p2085 was amplified using oligonucleotides pGFP-Inf-Prom-F and pGFP-vec-R (Table S2). pr852 was amplified from S. aureus DNA using oligonucleotides pGFP_852-Prom_fw and pGFP_852-Prom_rev. This fragment was cloned in the linearized vector using infusion cloning, transformed into E. coli DH5α, and plated on LB agar plates supplemented with 100 μg/ml ampicillin. The plasmid was isolated from E. coli, and the insert sequence was verified by Sanger sequencing, electroporated into S. aureus RN4220, and finally transferred to S. aureus 6850 by phage transduction.

### RNA-seq.

RNA was isolated from S. aureus using TRIzol as previously described (63). DNase I treatment was performed to remove remaining DNA. The concentration of the RNA was determined by spectrophotometry on a NanoDrop 1000 (Peqlab), and RNA integrity was examined using agarose gel containing formamide or by analysis via Bioanalyzer. Enrichment of mRNA was performed using the Universal Ribodepletion kit followed by Next Ultra Directional Library Preparation kit for Illumina (NEB). The cDNA was sequenced on a HiSeq 2500 (Illumina) yielding 100-bp paired-end reads. Adapters were removed using cutadapt (64), and only reads exceeding a mean base quality 5 within all sliding windows of 5 bp were mapped to the S. aureus 6850 genome (NCBI accession NC_022222 [65]). Read mapping was conducted using Bowtie2 (66). DESeq2 (60) was used to identify differentially regulated transcripts.

### qRT-PCR.

Reverse transcription of total isolated RNA was performed using RevertAID reverse transcriptase (Thermo Scientific). A 10-ng sample of cDNA was used to perform quantitative RT-PCR (qRT-PCR) in a one-step reaction using Sybr green master mix (Genaxxon) on a StepOne Plus real-time PCR system (Applied Biosystems). For primers used for qRT-PCR, see Table S2. Analysis was performed using the comparative threshold cycle (2^−ΔΔ^*^CT^*) method. Relative gene expression was normalized to expression of *gyrB* and to the corresponding expression under control conditions.

### Statistics.

Data were analyzed by Microsoft Excel and GraphPad Prism. Significance of differences from at least three independent replicates were determined by a Student’s *t* test for comparison of two independent data sets or with one-way analysis of variance (ANOVA) followed by Tukey’s multiple-comparison test for comparison of three independent data sets.
