# Supplementary figures and images for: Identification of a Novel LysR-Type Transcriptional Regulator in Staphylococcus aureus That Is Crucial for Secondary Tissue Colonization during Metastatic Bloodstream Infection
Source: mBio. 2020 Aug 25;11(4):e01646-20. doi: 10.1128/mBio.01646-20 (PMC7448277; doi:10.1128/mBio.01646-20)

Supplemental Figure 1

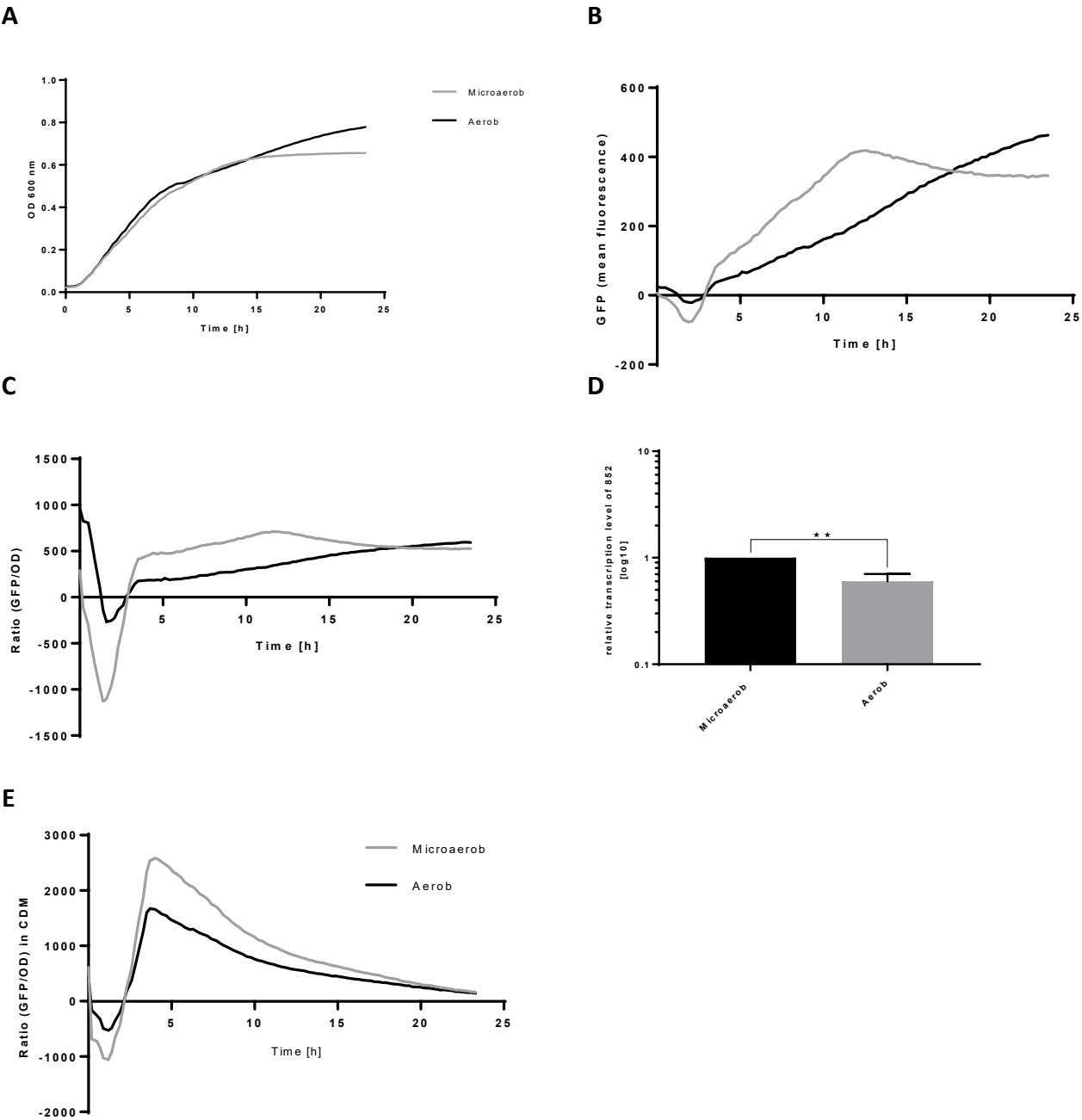

Supplement: FIG S1 [file mBio.01646-20-sf001.pdf]

Supplemental Figure 2

A

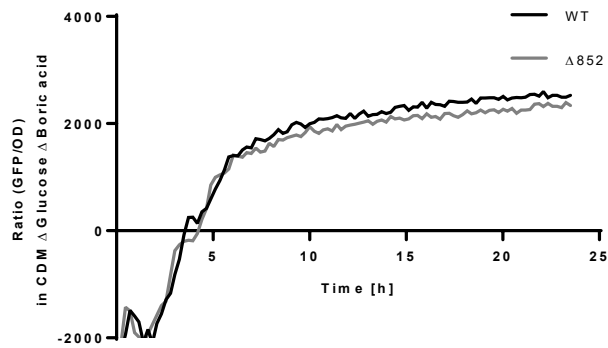

B

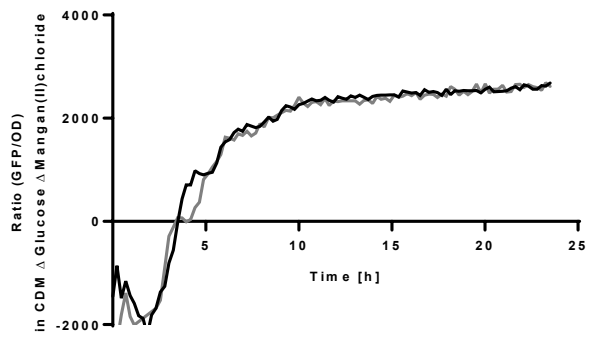

C

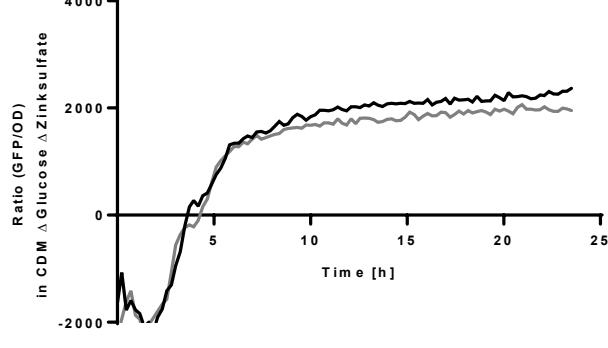

D

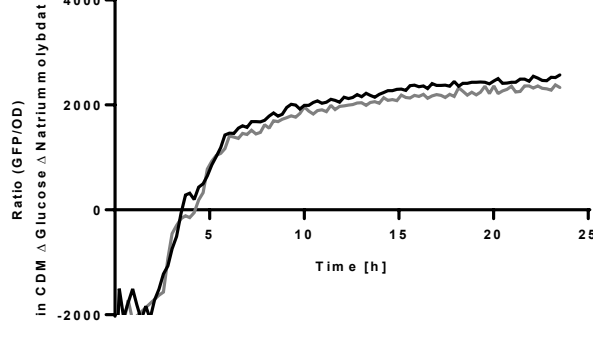

E

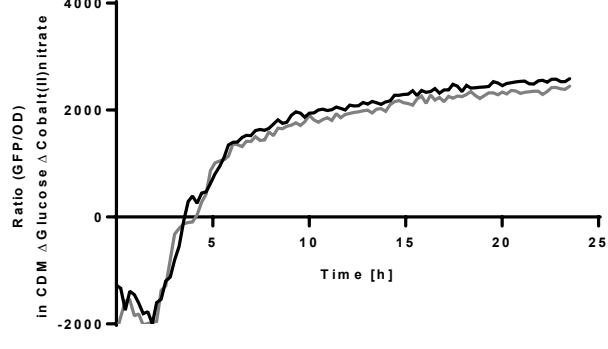

F

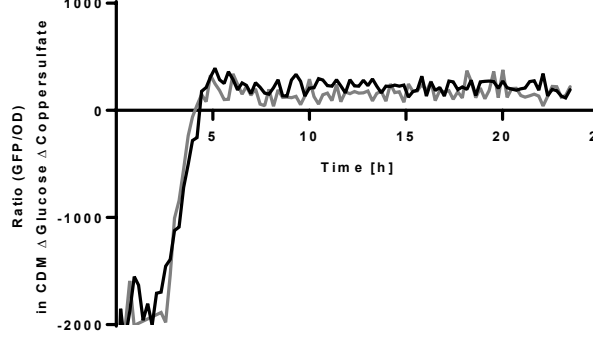

G

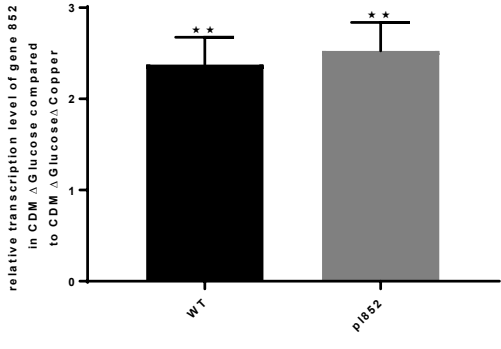

Supplement: FIG S2 [file mBio.01646-20-sf002.pdf]

# Supplemental Figure 3

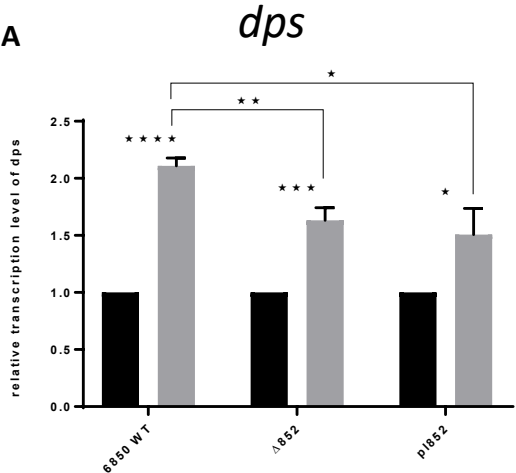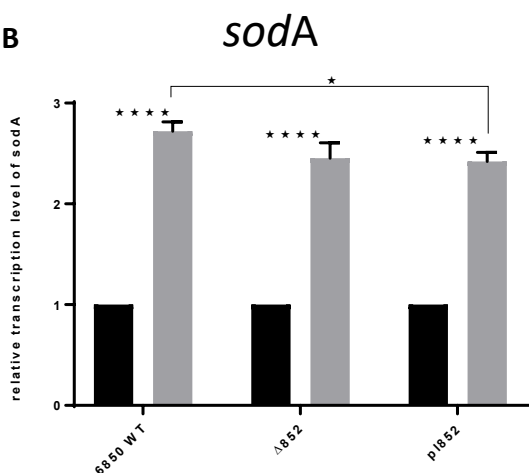

Supplement: FIG S3 [file mBio.01646-20-sf003.pdf]

# Supplemental Figure 4

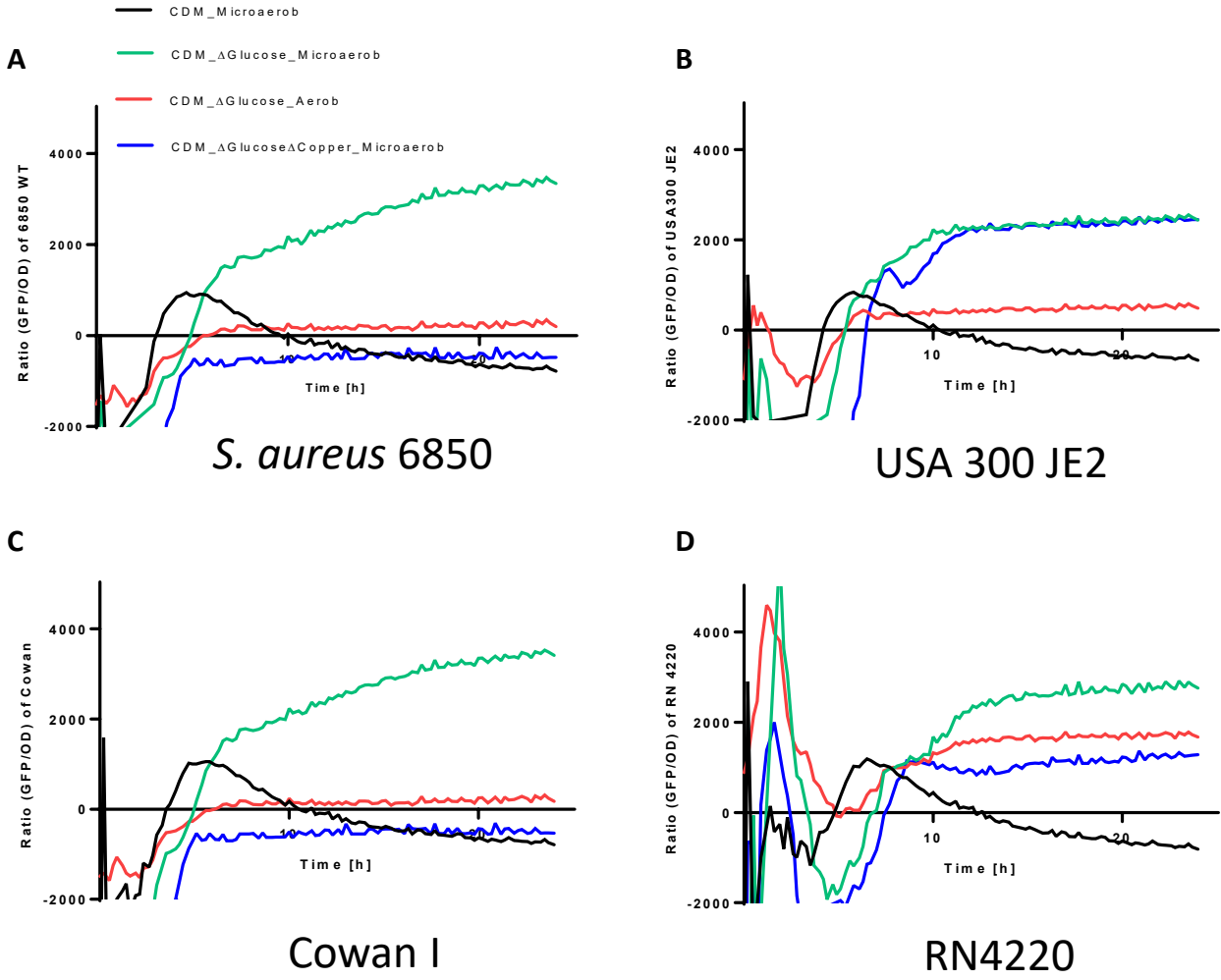

Supplement: FIG S4 [file mBio.01646-20-sf004.pdf]
