# Supplementary material for: Identification of a Novel LysR-Type Transcriptional Regulator in Staphylococcus aureus That Is Crucial for Secondary Tissue Colonization during Metastatic Bloodstream Infection
Source: mBio. 2020 Aug 25;11(4):e01646-20. doi: 10.1128/mBio.01646-20 (PMC7448277; doi:10.1128/mBio.01646-20)
Supplement: TABLE S1 [file mBio.01646-20-st001.pdf]

Table S1: Bacterial strains and plasmids used in this study

Strain	Description	Source
Escherichia coli		
DH5α	*fhuA2 lac(del)U169 phoA glnV44 Φ80' lacZ(del)M15 gyrA96 recA1 relA1 endA1 thi-1 hsdR17*	
Staphylococcus aureus		
RN4220	NCTC 8325-4 *sau1-, hsdR-,* laboratory strain accepting foreign DNA; β-toxin producer, non-hemolytic	Kreiswirth et al., 1983
6850	methicillin-sensitive; *spa* type t185, sequence type 50, Isolated from a patient with a skin abscess, progressed to bacteremia, osteomyelitis, septic arthritis, and multiple systemic abscesses	Ref. 19
6850 Δ852	*S. aureus* 6850 with deletion of RSAU_000852	This study
6850 pl852	*S. aureus* Δ852 complemented with pL852, Chloramphenicol-resistant	This study

Plasmid name	Description	Source
pl852	Expression plasmid for RSAU_000852 under control of its native promoter	This study
p2085	derivative of pALC2084 (Bateman et al., 2001)	Ref. 14
p2085-852	plasmid for anhydrous tetracycline-inducible expression of RSAU_000852	This study
p2085-Pr852-GFP	RSAU_000852 promoter fusion with green fluorescent protein (GFP)	This study

References:

- Kreiswirth BN, Lofdahl S, Betley MJ, O'Reilly M, Schlievert PM, Bergdoll MS, Novick RP. 1983. The toxic shock syndrome exotoxin structural gene is not detectably transmitted by a prophage. Nature 305:709-12. doi: 10.1038/305709a0
- Bateman BT, Donegan NP, Jarry TM, Palma M, Cheung AL. 2001. Evaluation of a tetracycline-inducible promoter in Staphylococcus aureus in vitro and in vivo and its application in demonstrating the role of sigB in microcolony formation. Infect Immun 69:7851-7.
