# Supplementary material for: Identification of a Novel LysR-Type Transcriptional Regulator in Staphylococcus aureus That Is Crucial for Secondary Tissue Colonization during Metastatic Bloodstream Infection
Source: mBio. 2020 Aug 25;11(4):e01646-20. doi: 10.1128/mBio.01646-20 (PMC7448277; doi:10.1128/mBio.01646-20)
Supplement: TABLE S2 [file mBio.01646-20-st002.pdf]

Table S2: Oligonucleotides used in this study

Name	Sequence (5´-3´)
RT-PCR	
**RT-852-F**	TGAACAATATGAAAGTGGCA
**RT-852-R**	GGATGCTCGTTAAAGAATG
**RT-copA-F**	CGCTGTAGAAACTGTCGAATTAG
**RT-copA-R**	GGATAATAGTCAACTTTAGCTTGC
**RT-copB-F**	GTTGAAGGTATGAGCTGTGG
**RT-copB-R**	CGACATCGTAACCTTGATCTTC
**RT-gabT-F**	GTATCATGAACCGTTGGTACG
**RT-gabT-R**	GAGCCAAAAGTTGAACCATG
**RT-ilvA-F**	CATGTGCGAGTGCAGG
**RT-ilvA-R**	CAGTGATCAAATGTATCACCAGTG
**RT-ilvB-F**	GAACAAGGTGCTGTTCATGC
**RT-ilvB-R**	CTTGTCCGGTGAATACAACTAG
**RT-ilvC-F**	TGAAACATTAGTAGAAGCGG
**RT-ilvC-R**	CGTGGTCCTGAAACATAGTC
**RT-ilvD-F**	GGAATAGCTATGGGACATATCG
**RT-ilvD-R**	CAGAGCAAAAGATAGCTGGTAC
**RT-ilvH-F**	CCCGGGATTTCTAACATGG
**RT-ilvH-R**	GGCAGATGTTAACTAGCAGC
**RT-lacC-F**	GATCATGCCGGCATCAAG
**RT-lacC-R**	CCTGAAATAGCAACTGCTTC
**RT-lacE-F**	CTATACGATGGGGCTTGTAGC
**RT-lacE-R**	CCCATAAATGCACTTAAGAATCC
**RT-leuA2-F**	GGGATGGTATGCAACAAAGTAATGT
**RT-leuA2-R**	GGACTTAAAATGTCTCTGAATTGATG
**RT-leuB-F**	GCAATCGGTGGACCTAAATG
**RT-leuB-R**	CACGGACTATAACTAAATCTGTACC
**RT-leuC-F**	CGCCATAGATTTTGGGGTG
**RT-leuC-R**	GTTGCGAAAACATGTTCAACTTC
**RT-lrgA-F**	CTGGTGCTGTTAAGTTAGGC
**RT-lrgA-R**	GTATTGTTGAGACGATTATTAGTCC
**RT-msrA-F**	GGACCACGGTCTTGATATTG
**RT-msrA-R**	GGCGGACATATTGAAAATCC
**RT-purQ-F**	CTGAAGGTAAGCCAGTATTAGG
**RT-purQ-R**	CCGTGAGCTACAGGATATATAAC
**RT-pyrAB-F**	GAGCAACCTGACGCTTTAC
**RT-pyrAB-R**	GTTCTAAACATTTCACGGTCTTC
**RT-pyrB-F**	CGAATATTAACATCCCAATTGCG
**RT-pyrB-R**	GCACCTAATGCTTTTAAACTATGG
**RT-pyrC-F**	GACAATTGAAACTGGTACTAAAGC
**RT-pyrC-R**	CCTAATTGACGTGTTGTAATTGAAG
**RT-RNAIII-F**	ACATAGCACTGAGTCCAAGG
**RT-RNAIII-R**	TCGACACAGTGAACAAATTC
**RT-ureB-F**	CAGAGGTTGAAATTAATAACCAT
**RT-ureB-R**	CTCCAGCTGGAATATCTAAATG
**RT-ureC-F**	GCAGACCTTGTTATTTCTAATGC
**RT-ureC-R**	CAATACCACCAGCAGTGA
Cloning	
**852-BamHI-R**	TAATTGGATCCTTATAATTGTTCATTGGCAATATACTT
**852-NotI-F**	AAATAGCGGCCGCCGAACATTACTTTGTTGCATAC
**852_test_R**	TATCTCCATACAATTTCCAATC
**852_test_F**	AAGCGATTGATTTAGTTGAC
**attB2-852-down-R**	GGGGACCACTTTGTACAAGAAAGCTGGGTGTTTAAAT ATATTTTCACCAATTATAGGTTTG
**852-down-F-SacII**	GATCGACCGCGGGAACTTACCTCTTTCAAAAAAGTTAATAATT
**852-up-R-SacII**	GATCGACCGCGGAATAAATTTCAAATCTAAAAAACCAAGAATGC
**attB1-852-up-F**	GGGGACAAGTTTGTACAAAAAAGCAGGCTATAAACGTGTT GTAGGTCAAGATAAA
**pGFP-Inf-Prom F**	GAATTCTTAGGAGGATGATTATTTATGAGTAAAGGAGAAGAAC
**pGFP vec R**	GCATGCAAGCTTTTAAAAAGCAAATATGAGCCAAATAAA
**pGFP_852-Prom_fw**	TAAAAGCTTGCATGCGATATTTTGAAATAATTTC
