# Supplementary material for: Identification of a Novel LysR-Type Transcriptional Regulator in Staphylococcus aureus That Is Crucial for Secondary Tissue Colonization during Metastatic Bloodstream Infection
Source: mBio. 2020 Aug 25;11(4):e01646-20. doi: 10.1128/mBio.01646-20 (PMC7448277; doi:10.1128/mBio.01646-20)
Supplement: TABLE S3 [file mBio.01646-20-st003.pdf]

Table S3: Enrichment in GO-terms as determined by STRING-DB.org

GO-TERM (BIOLOGICAL PROCESS)	DESCRIPTION	FDR
**GO:0009082**	**branched-chain amino acid biosynthetic process**	**9.14e-06**
**GO:0009081**	branched-chain amino acid metabolic process	9.14e-06
**GO:1901607**	alpha-amino acid biosynthetic process	9.45e-05
**GO:1901605**	alpha-amino acid metabolic process	9.45e-05
**GO:0044281**	small molecule metabolic process	9.45e-05
**GO:0019627**	**urea metabolic process**	**9.45e-05**
**GO:0006520**	cellular amino acid metabolic process	9.45e-05
**GO:0008652**	cellular amino acid biosynthetic process	0.00010
**GO:0009097**	isoleucine biosynthetic process	0.00031
**GO:0006549**	isoleucine metabolic process	0.00031
**GO:1901564**	organonitrogen compound metabolic process	0.0013
**GO:0044283**	small molecule biosynthetic process	0.0013
**GO:0009987**	cellular process	0.0020
**GO:0008152**	metabolic process	0.0024
**GO:0006807**	nitrogen compound metabolic process	0.0042
**GO:0009099**	valine biosynthetic process	0.0046
**GO:0044205**	**'de novo' UMP biosynthetic process**	**0.0072**
**GO:0043419**	urea catabolic process	0.0072
**GO:0006573**	valine metabolic process	0.0072
**GO:1901566**	organonitrogen compound biosynthetic process	0.0081
**GO:0044237**	cellular metabolic process	0.0081
**GO:0009098**	leucine biosynthetic process	0.0086
**GO:0006551**	leucine metabolic process	0.0086
**GO:0071704**	organic substance metabolic process	0.0111
**GO:0046049**	UMP metabolic process	0.0111
**GO:0046132**	pyrimidine ribonucleoside biosynthetic process	0.0191
**GO:0046131**	pyrimidine ribonucleoside metabolic process	0.0191
**GO:0009156**	ribonucleoside monophosphate biosynthetic process	0.0191
**GO:0006812**	cation transport	0.0253
**GO:0009260**	ribonucleotide biosynthetic process	0.0284
**GO:0009161**	ribonucleoside monophosphate metabolic process	0.0284
**GO:0044238**	primary metabolic process	0.0298
**GO:0019637**	organophosphate metabolic process	0.0327
**GO:0019835**	cytolysis	0.0339
**GO:0044249**	cellular biosynthetic process	0.0379
**GO:0009259**	ribonucleotide metabolic process	0.0392
**GO:0009064**	glutamine family amino acid metabolic process	0.0396
**GO:0044248**	cellular catabolic process	0.0412
