# Supplementary material for: Identification of a Novel LysR-Type Transcriptional Regulator in Staphylococcus aureus That Is Crucial for Secondary Tissue Colonization during Metastatic Bloodstream Infection
Source: mBio. 2020 Aug 25;11(4):e01646-20. doi: 10.1128/mBio.01646-20 (PMC7448277; doi:10.1128/mBio.01646-20)
Supplement: TABLE S4 [file mBio.01646-20-st004.pdf]

Table S4. Gene mutants identified enriched or depleted in murine liver after intravenous infection (cutoff: adj. p-Values <= 0.0005)

ID	log_2FC	Adj. **p-Value**	gene	annotation
**RSAU_001097**	-5.74	0.0000233	def	Peptide deformylase
**RSAU_000902**	-5.35	0.0000354	ugtP	Processive diacylglycerol glucosyltransferase
**RSAU_000586**	-5.26	7.42E-07	rsaC	non-coding RNA RsaC
**RSAU_000571**	-4.64	8.67E-08		Putative membrane protein
**RSAU_001052**	-3.57	0.0000599	eta	C4-dicarboxylate transporter/malic acid transport family protein
**RSAU_000958**	-3.22	0.00002	purM	Phosphoribosylformylglycinamidine cyclo-ligase
**RSAU_002404**	-3.19	0.0000223	crtQ	4,4'-diaponeurosporenoate glycosyltransferase
**RSAU_000985**	-3.02	0.000135	potB	Spermidine/putrescine ABC transporter permease protein
**RSAU_002542**	-2.79	0.0000219		DNA-binding protein, putative
**RSAU_000940**	3.44	0.000137	atl	Bifunctional autolysin / N-acetylmuramoyl-L-alanine amidase
**RSAU_002096**	3.74	5.78E-07	pycA	RND multidrug transporter; Acriflavin resistance protein
**RSAU_001615**	3.83	0.000134	leuS	Leucyl-tRNA synthetase
**RSAU_001480**	3.99	0.000131	alaS	Alanyl-tRNA synthetase
**RSAU_000981**	4.05	0.0000396	pdhD	Dihydrolipoamide dehydrogenase
**RSAU_000014**	4.06	0.0000659		Phosphoesterase, DHH family protein
**RSAU_002342**	4.07	0.00002	gntP	Gluconate permease
**RSAU_002402**	4.18	0.000123	crtN	Squalene/phytoene synthase
**RSAU_002410**	4.21	0.000084	isaA	Immunodominant antigen A, transglycosylase isaA
**RSAU_000222**	4.22	0.00000207	lytM	Peptidoglycan hydrolase
**RSAU_000494**	4.22	0.00000151	rpoB	DNA-directed RNA polymerase subunit beta
**RSAU_000758**	4.25	0.0000938	rnr	Ribonuclease R
**RSAU_001970**	4.27	0.0000804		ATP-GRASP domain protein
**RSAU_002274**	4.34	0.0000386	bcr	Drug resistance transporter protein
**RSAU_001152**	4.34	0.000135	infB	Translation initiation factor IF-2
**RSAU_002241**	4.41	0.0000219	fnt	Formate/nitrite transporter family protein
**RSAU_000888**	4.41	0.000196		Monovalent cation/H+ antiporter-2 family protein
**RSAU_002255**	4.5	0.000154	gpmA	2,3-bisphosphoglycerate-dependent phosphoglycerate mutase
**RSAU_001560**	4.51	0.0000317	dnaE	DNA polymerase III subunit alpha
**RSAU_000065**	4.53	0.0000518		hypothetical protein
**RSAU_002302**	4.62	0.0000219	opp-1D	Oligopeptide ABC transporter, ATPase domain, putative
**RSAU_000266**	4.63	0.000151	geh	glycerol ester hydrolase
**RSAU_001210**	4.63	0.0000451	dhoM	Homoserine dehydrogenase
**RSAU_001882**	4.68	0.0000219	gcp	Putative DNA-binding/iron metalloprotein/AP endonuclease
**RSAU_000168**	4.7	0.00018	walK	Sensor histidine kinase protein
**RSAU_000943**	4.75	0.0000599	nanE	Cell envelope-related transcriptional attenuator domain protein
**RSAU_002525**	4.83	0.00014	hisZ	ATP phosphoribosyltransferase regulatory subunit
**RSAU_002298**	4.89	0.000149	gltB2	Ferredoxin-dependent glutamate synthase
**RSAU_000666**	4.91	0.0000599		Transporter Anion:Sodium Symporter family protein
**RSAU_001351**	4.94	0.0000354	ebpS	Elastin binding protein
**RSAU_002089**	4.96	0.0000023	pbuG	Xanthine/uracil permease family protein
**RSAU_002272**	4.96	0.000123	glxK	Glycerate kinase

**RSAU_001253**	4.98	0.00000748	trpB	Tryptophan synthase beta chain
**RSAU_002290**	4.99	0.000137		Amino acid transporter permease
**RSAU_000117**	5.06	0.000125	capO	Capsular polysaccharide synthesis UDP-N-acetyl-D dehydrogenase
**RSAU_000844**	5.13	0.00000323	addA	ATP-dependent helicase/nuclease subunit A
**RSAU_002453**	5.13	0.0000023	gbsA	Glycine betaine aldehyde dehydrogenase
**RSAU_001518**	5.14	0.000166		hypothetical protein
**RSAU_000906**	5.15	0.000193	terC	Toxic anion resistance membrane protein
**RSAU_000937**	5.15	0.0000712	aspC	Putative aminotransferase
**RSAU_001219**	5.18	0.000052	guaC	Guanosine monophosphate reductase
**RSAU_000592**	5.19	0.000123	tagA	Teichoic acid biosynthesis protein
**RSAU_000600**	5.19	0.000162	nupC2	Na+ dependent nucleoside transporter protein
**RSAU_001943**	5.19	0.000135	atpA	FOF1 ATP synthase subunit alpha
**RSAU_000994**	5.22	5.78E-07	typA	GTP-binding protein TypA/BipA
**RSAU_002485**	5.22	0.0000599	pmi	Mannose-6-phosphate isomerase
**RSAU_001860**	5.24	0.00000798	groEL	60 kDa chaperonin GroEL
**RSAU_002334**	5.24	0.0000451	pgcA	Phosphoglucomutase/phosphomannomutase
**RSAU_000265**	5.28	0.0000283	sirA	Nucleoside recognition domain protein
**RSAU_002372**	5.32	0.000156	sdaAA	L-serine dehydratase, iron-sulfur-dependent, alpha subunit
**RSAU_002291**	5.34	0.0000668	pnbA	Para-nitrobenzyl esterase chain A
**RSAU_000450**	5.36	0.000123	ftsH	Cell division protein FtsH, putative
**RSAU_000794**	5.37	0.0000651	sufS	Cysteine desulfurase, SufS subfamily
**RSAU_001157**	5.39	0.00000436	pnpA	Polyribonucleotide nucleotidyltransferase
**RSAU_002278**	5.45	0.000162	cycA	Amino acid permease, putative
**RSAU_002357**	5.45	0.00013	fbp	Fructose-1,6-bisphosphatase class 3
**RSAU_001388**	5.49	0.0000124	ispA	Geranyltranstransferase
**RSAU_000974**	5.51	0.000032	rnjA	Ribonuclease J 1
**RSAU_001991**	5.55	0.0000451	glmS	Glucosamine--fructose-6-phosphate aminotransferase
**RSAU_002484**	5.65	0.0000389	manP	PTS system, fructose-specific IIABC component
**RSAU_002100**	5.65	0.000128		Major facilitator transporter
**RSAU_000062**	5.69	0.00000243		67 kDa myosin-cross-reactive antigen
**RSAU_001080**	5.69	0.0000932	rluD	Ribosomal large subunit pseudouridine synthase
**RSAU_001786**	5.71	0.00000788	gatB	Aspartyl/glutamyl-tRNA amidotransferase subunit B
**RSAU_000693**	5.72	0.0000599	ltaS	Glycerol phosphate lipoteichoic acid synthase
**RSAU_002444**	5.72	0.00000207		Amino acid permease family protein
**RSAU_000739**	5.75	0.0000733	trxB	Thioredoxin reductase
**RSAU_001145**	5.82	0.0000497	proS	Prolyl-tRNA synthetase
**RSAU_000718**	5.86	7.42E-07	pepT	Peptidase T
**RSAU_000864**	5.88	0.000117	oppA	Oligopeptide ABC transporter-binding protein OppA
**RSAU_000833**	5.9	0.000135	cls	Family NADH-dependent flavin oxidoreductase, putative
**RSAU_002020**	5.96	0.0000606	htsB	Iron ABC transporter, permease protein
**RSAU_000289**	5.99	0.00000791	efeB	Dyp-type peroxidase family protein
**RSAU_002289**	6	0.00000748	rpsP	Bacteriocin-protection, YdeI/OmpD-Associated family protein
**RSAU_002442**	6.05	0.000137	budA	Alpha-acetolactate decarboxylase
**RSAU_000420**	6.12	0.000159	ribA	Protein from nitrogen regulatory protein P-II family YAAQ
**RSAU_001032**	6.12	0.00000436	sdhA	Succinate dehydrogenase flavoprotein subunit
**RSAU_001394**	6.14	0.0000233	accB	Biotin carboxyl carrier protein of acetyl-CoA carboxylase

**RSAU_002041**	6.14	0.0000466	cobB	NAD-dependent protein deacetylase SIR2 family
**RSAU_001189**	6.18	0.000117	hflX	GTP-binding protein HflX
**RSAU_002276**	6.31	5.91E-07		Peptidase C39 like family protein
**RSAU_002432**	6.32	0.000126		Adenine nucleotide alpha hydrolases superfamily protein
**RSAU_000300**	6.47	0.0000194		Cyclase family protein
**RSAU_002165**	6.5	3.46E-07	hipO2	N-L-amino acid amidohydrolase
**RSAU_000093**	6.54	0.0000219	drm	Phosphopentomutase
**RSAU_000497**	6.56	0.000195	rpsL	30S ribosomal protein S12
**RSAU_001643**	6.56	0.0000213	metK	S-adenosylmethionine synthetase
**RSAU_001034**	6.69	0.0000451	murI	Glutamate racemase
**RSAU_000566**	6.84	0.0000314	btuF	Iron ABC transporter binding protein
**RSAU_000534**	6.92	0.000117		putative Membrane protein
**RSAU_000505**	7	0.0000147	capD	NAD dependent epimerase/dehydratase family protein
**RSAU_000506**	7.03	0.0000354	ilvE	Branched-chain amino acid aminotransferase
**RSAU_001527**	7.03	0.0000512	hemB	Delta-aminolevulinic acid dehydratase
**RSAU_000498**	7.25	0.000117	rpsG	30S ribosomal protein S7
**RSAU_000427**	7.38	0.00000243	metG	Methionyl-tRNA synthetase
**RSAU_002292**	7.91	1.21E-09	rimM	Major facilitator transporter, chloramphenicol
