# Supplementary material for: Identification of a Novel LysR-Type Transcriptional Regulator in Staphylococcus aureus That Is Crucial for Secondary Tissue Colonization during Metastatic Bloodstream Infection
Source: mBio. 2020 Aug 25;11(4):e01646-20. doi: 10.1128/mBio.01646-20 (PMC7448277; doi:10.1128/mBio.01646-20)
Supplement: TABLE S5 [file mBio.01646-20-st005.pdf]

Table S5: *S. aureus* transposon mutants found either enriched or depleted in both, kidney or liver of mice after intravenous infection by TN-seq. Counts of sequenced transposon insertion sites were compared with the counts obtained from the infection inoculum. By comparing this inocolum to the recovered samples genes which are lost in a specific tissue are represented by negative $log2_FC$ and vice versa.

6850 Locus ID	NCTC8325 Locus ID	Gene	Annotation	log2FC Inoc./ Kidney	Adj. p-Value Kidney (<1e-3)	logFC Inoc./ Liver	Adj. p-Value Liver (<1e-3)
**RSAU_000958**	SAOUHSC_01015	purM	Phosphoribosylformyl glycinamidine cyclo-ligase	-5	0.00015	-3.22	0.00002
**RSAU_000571**	SAOUHSC_00618		Putative membrane protein	-4.04	0.000124	-4.64	8.67E-08
**RSAU_002542**	SAOUHSC_03032		DNA-binding protein, putative	-2.94	0.000124	-2.79	0.0000219
**RSAU_000494**	SAOUHSC_00524	rpoB	DNA-directed RNA polymerase subunit beta	3.72	0.000275	4.22	0.00000151
**RSAU_000940**	SAOUHSC_00994	atl	Bifunctional autolysin / N-acetylmuramoyl-L-alanine amidase / Endo-N	3.74	0.00024	3.44	0.000137
**RSAU_000222**	SAOUHSC_00248	lytM	Peptidoglycan hydrolase	3.96	0.000124	4.22	0.00000207
**RSAU_000981**	SAOUHSC_01043	pdhD	Dihydrolipoamide dehydrogenase	4.09	0.000285	4.05	0.0000396
**RSAU_001882**	SAOUHSC_02277	gcp	Putative DNA-binding/iron metalloprotein/AP endonuclease	4.26	0.000388	4.68	0.0000219
**RSAU_001970**	SAOUHSC_02373		ATP-grasp domain protein	4.4	0.000262	4.27	0.0000804
**RSAU_002089**	SAOUHSC_02516	pbuG	Xanthine/uracil permease family protein	4.43	0.000202	4.96	0.0000023
**RSAU_000495**	SAOUHSC_00525	rpoC	DNA-directed RNA polymerase subunit beta'	4.5	0.000774	4.41	0.000206
**RSAU_000943**	SAOUHSC_00997	nanE	Cell envelope-related transcriptional attenuator domain protein	4.53	0.000632	4.75	0.0000599
**RSAU_002274**	SAOUHSC_02725	bcr	Drug resistance transporter protein	4.53	0.000189	4.34	0.0000386
**RSAU_000822**	SAOUHSC_00882		Pyridine nucleotide-disulfide oxidoreductase family protein	4.57	0.000291	3.95	0.000453
**RSAU_001560**	SAOUHSC_01811	dnaE	DNA polymerase III subunit alpha	4.81	0.000124	4.51	0.0000317
**RSAU_000844**	SAOUHSC_00905	addA	ATP-dependent helicase/nuclease subunit A	4.84	0.000124	5.13	0.00000323
**RSAU_002334**	SAOUHSC_02793	pgcA	Phosphoglucomutase / phosphomannomutase	5	0.000783	5.24	0.0000451
**RSAU_000735**	SAOUHSC_00781	hprK	HPr kinase/phosphorylase	5.04	0.00081	4.64	0.000453
**RSAU_000193**	SAOUHSC_00217	gutB	Zinc-binding sorbitol dehydrogenase	5.1	0.000236	4.2	0.000667
**RSAU_001351**	SAOUHSC_01501	ebpS	Elastin binding protein	5.14	0.000124	4.94	0.0000354
**RSAU_002484**	SAOUHSC_02975	manP	PTS system, fructose-specific IIABC component	5.15	0.000555	5.65	0.0000389
**RSAU_002525**	SAOUHSC_03015	hisZ	ATP phosphoribosyltransferase regulatory subunit	5.16	0.000305	4.83	0.00014
**RSAU_000259**	SAOUHSC_00293	nupC	Nucleoside transporter permease	5.17	0.00092	5	0.000229
**RSAU_000009**	SAOUHSC_00009	serS	Seryl-tRNA synthetase	5.19	0.000124	4.07	0.000819
**RSAU_000666**	SAOUHSC_00698		Transporter Anion:Sodium Symporter family protein	5.22	0.00015	4.91	0.0000599
**RSAU_001219**	SAOUHSC_01330	guaC	Guanosine monophosphate reductase	5.28	0.00025	5.18	0.000052
**RSAU_000600**	SAOUHSC_00648	nupC2	Na+ dependent nucleoside transporter protein	5.31	0.000774	5.19	0.000162
**RSAU_002444**	SAOUHSC_02923		Amino acid permease family protein	5.35	0.000117	5.72	0.00000207
**RSAU_000994**	SAOUHSC_01058	typA	GTP-binding protein TypA/BipA	5.58	0.000000408	5.22	0.000000578

**RSAU_002289**	SAOUHSC_02747	rpsP	Bacteriocin-protection, YdeI/OmpD-Associated family protein	5.6	0.000196	6	0.00000748
**RSAU_001080**	SAOUHSC_01163	rluD	Ribosomal large subunit pseudouridine synthase	5.62	0.000742	5.69	0.0000932
**RSAU_002278**	SAOUHSC_02729	cycA	Amino acid permease, putative	5.7	0.000486	5.45	0.000162
**RSAU_000062**	SAOUHSC_00061		67 kDa myosin-cross-reactive antigen	5.76	0.0000324	5.69	0.00000243
**RSAU_001145**	SAOUHSC_01240	proS	Prolyl-tRNA synthetase	5.77	0.000291	5.82	0.0000497
**RSAU_000638**	SAOUHSC_00668	vraG	ABC transporter permease	5.94	0.000124	5.02	0.000259
**RSAU_000864**	SAOUHSC_00927	oppA	Oligopeptide ABC transporter-binding protein OppA	5.97	0.000422	5.88	0.000117
**RSAU_002020**	SAOUHSC_02428	htsB	Iron ABC transporter, permease protein	6.21	0.000202	5.96	0.0000606
**RSAU_000696**	SAOUHSC_00731		Glycine betaine/choline ABC transporter ATP-binding protein	6.23	0.000521	5.48	0.000688
**RSAU_000427**	SAOUHSC_00461	metG	Methionyl-tRNA synthetase	6.5	0.00024	7.38	0.00000243
**RSAU_002432**	SAOUHSC_02911		Adenine nucleotide alpha hydrolases superfamily protein	6.6	0.000309	6.32	0.000126
**RSAU_002292**	SAOUHSC_02752	rimM	Major facilitator transporter, chloramphenicol	7.11	0.000000322	7.91	1.21E-09
**RSAU_000505**	SAOUHSC_00535	capD	NAD dependent epimerase/dehydratase family protein	7.2	0.0000655	7	0.0000147
